# Forward modelling the rubber hand: illusion of ownership modifies motor-sensory predictions by the brain

**DOI:** 10.1098/rsos.160407

**Published:** 2016-08-24

**Authors:** Laura Aymerich-Franch, Damien Petit, Abderrahmane Kheddar, Gowrishankar Ganesh

**Affiliations:** 1 CNRS-AIST Joint Robotics Laboratory (JRL), UMI3218/RL, Tsukuba, Japan; 2 CNRS-UM LIRMM, Interactive Digital Human group, UMR5506, Montpellier, France

**Keywords:** rubber-hand illusion, forward model, body ownership

## Abstract

The question of how we attribute observed body parts as our own, and the consequences of this attribution on our sensory-motor processes, is fundamental to understand how our brain distinguishes between self and other. Previous studies have identified interactions between the illusion of ownership, and multi-sensory integration and cross-sensory predictions by the brain. Here we show that illusory ownership additionally modifies the motor-sensory predictions by the brain. In our preliminary experiments, we observed a new numbness illusion following the classical rubber-hand illusion (RHI); brushing only the rubber hand after induction of the RHI results in illusory numbness in one's real hand. Previous studies have shown that self-generated actions (like tickling) are attenuated by motor-sensory predictions by the so-called *forward model*. Motivated by this finding, here we examined whether the numbness illusion after the RHI is different when the rubber hand is brushed oneself, compared with when the brushing is performed by another. We observed that, all other conditions remaining the same, haptic perception in the real hand was lower (numbness higher) during self-generated brushing. Our result suggests that RHI reorganizes the forward model, such that we predict haptic consequences of self-generated motor actions on the rubber hand.

## 1. Introduction

Embodiment of limbs and the associated ownership are largely agreed to be a consequence of multi-sensory integration in the brain [1–5]. It is believed that embodiment and illusory ownership additionally influence multi-sensory predictions [6–9], resulting in illusory sensations in their real hand or body when subjects observe stimulations of the embodied rubber hand [10,11] or avatar [8,9]. Little is known, however, about whether the illusion of ownership also influences the *motor*-sensory predictions by the so-called *forward models* in the brain.

Forward models refer to the neural circuitry implicated in predicting sensory outcomes of self-generated movements [12]. These predictions are fundamental to our motor abilities and play crucial roles in perception of self-generated actions [13–16], online motor control [17] and motor learning [18]. The motor-sensory predictions from forward models are also known to modulate haptic perception in individuals resulting in differences between perceptions of self-induced haptic stimulations and those induced by others [19–23]—this, for example, is said to explain why we are tickled by others, but cannot tickle ourselves [22]. Here, we exploit this observation to evaluate whether the rubber-hand illusion (RHI) reorganizes the forward model in the brain.

In preliminary experiments in our laboratory we discovered that, following induction of the RHI, brushing only the rubber hand results in illusory numbness in the subject's real hand. This is a vivid (but previously unreported) illusion that subjects anecdotally reported as ‘my real hand feels dead’, ‘I feel disconnected in sensation from the real hand’ and ‘it seems like after I sat on my real hand’. Whereas physiological numbness, most often accompanied with tingling, is generally due to carpal tunnel syndrome, peripheral neuropathy or pinched nerves, the numbness illusion here is probably due to the haptic predictions corresponding to the vision of the rubber hand being touched, similar to reports of other multi-sensory predictions during ownership illusions [10,11]. In the presence of these predictions, the absence of real haptic feedback is perceived by subjects as numbness in their real hand. In order to investigate whether the forward model is affected during the RHI, here we examined how the illusory numbness after RHI is modulated when the brushing is performed by another individual, compared with when the brushing is self-generated.

Specifically, it is believed that haptic perception in humans is a consequence of the ‘subtraction’, from the real haptic signals, of the action induced haptic prediction by the forward model [12,14]. We thus hypothesized that if, in addition to the multi-sensory predictions, the motor-sensory predictions are modified by the RHI, then a self-generated touch of the rubber hand would induce a prediction of haptic feedback from the rubber hand. This additional prediction of haptic sensation from the rubber hand, when subtracted from the (absent) tactile signals when the rubber hand is brushed, would further decrease the haptic perception from the real hand. In other words, we hypothesized that if the forward model is reorganized by the RHI, then the illusory numbness during the brushing of an embodied rubber hand would be *more* when the brushing is self-generated, compared with when the same brushing is performed by another individual. We performed two RHI experiments to verify this hypothesis.

## 2. Experiment 1

**Figure 1.**
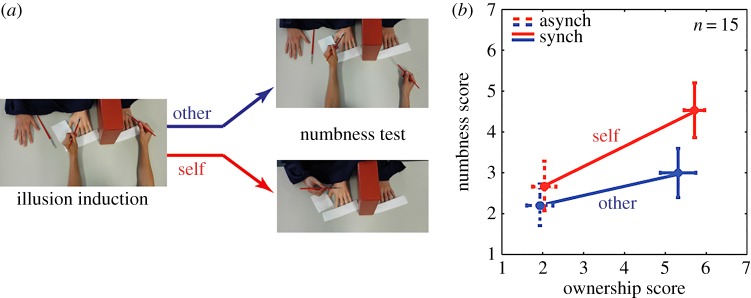
Experiment 1. (*a*) The experimenter started each condition by brushing the left hand of the subjects and a rubber hand, either synchronously or asynchronously (as control). This was followed by the numbness test where the subjects either watched only the rubber hand being brushed by an experimenter (other condition) or brushed the rubber hand themselves (self condition). (*b*) The numbness score and the ownership score on seven-point Likert scales plotted together. A 2 × 2 ANOVA revealed that, while the ownership did not change between the *self* and *other* conditions, the numbness changed significantly, *F*_1,56_ = 4.576, *p *= 0.037.

Experiment 1 included 15 subjects. All subjects experienced four experimental conditions: synchronous (synch) and asynchronous (asynch) brushing of the rubber hand and the real hand, followed by either *self* or *other* (generated) brushing of only the rubber hand. The order of the presented conditions was randomized across subjects (see the electronic supplementary material for details). All conditions started with an experimenter brushing the participants' visually occluded left hand, and a visible left rubber hand, either synchronously or asynchronously (figure 1*a*). The brush strokes were applied (not at one location but) on all the fingers and the back of hand in a random order such that the same finger or hand area were stroked at any time in the synch conditions on both the rubber hand and the real left hand. The brush strokes in the asynch conditions were asynchronous both in stroke time and in terms of stroked area. The brush strokes were performed for 90 s after which the subjects were asked to verbally answer (on a seven-point Likert scale) three *ownership questions* (see measures in the electronic supplementary material) in order to evaluate the ownership of the rubber hand. After the questionnaire, the brushing was continued for 30 s. Following this, in the *other* conditions, the experimenter stopped brushing the real hand and brushed only the rubber hand (see ‘numbness test’ in figure 1*a*), whereas in the *self* conditions, the experimenter stopped brushing both hands and the subjects were asked to pick up the brush with their right hand and brush the rubber hand themselves. A few seconds after the (only) rubber-hand brushing started, subjects were asked to rate any perceived *numbness* (explained as the hand feeling ‘sense-less’ or ‘dead’) in their left hand on a seven-point Likert scale (see the electronic supplementary material for ratings of other sensations), while they continued to watch the rubber hand being brushed.

We first compared the reported perception of embodiment across our experiment (measured as the average score of the embodiment questionnaire, see the electronic supplementary material). Because some groups of data did not conform to a normal distribution (Shapiro–Wilk test of normality *p* ≤ 0.05), we used the aligned rank transform (ART) for non-parametric factorial data analysis for a pre-processing step to align data before applying averaged ranks [24]. After which, we conducted a 2 × 2 within subject ANOVA. The first factor was the visuo-tactile brushing synchrony, which had two levels: synchronous and asynchronous. The second factor was brushing type, which was either *other* or *self.* We observed a significant main effect of the visuo-tactile synchrony, *F*_1,56_ = 102.096, *p *<* *0.0001, but no main effect in regard to the brushing type, *F*_1,56_ = 0.646, *p *= 0.425. No interaction was observed between the two factors, *F*_1,56_ = 0.415, *p *= 0.839. Thus, while the RHI increased during synchronous brushing, it was similar between the *self* and *other* conditions. Next, the numbness was compared between the same two factors in a separate 2 × 2 ANOVA. Again, because some groups of data were not normally distributed (Shapiro–Wilk test of normality *p* ≤ 0.05), we used the ART before the ANOVA. For numbness, we observed a significant main effect for both visuo-tactile synchrony, *F*_1,56_ = 6.059, *p *= 0.017, and brushing type, *F*_1,56_ = 4.576, *p *= 0.037. However, contrary to our expectation, the interaction between the two factors did not reach significance, *F*_1,56_ = 1.647, *p *= 0.205.

To check that this lack of interaction was not due to the individual differences in ownership change during the *other* and *self* conditions (as also suggested from one of the low ownership *F* value), we next checked how the numbness change in individuals varied with their reported ownership. We modelled the numbness change (Δ*N*) between the asynch and synch conditions to depend linearly on the ownership change (Δ*O*) between the same two conditions, Δ*N* =*β *× Δ*O*. We then calculated the *numbness change per unit ownership change* (given by *β*) for each subject and compared how *β* differed between the *self* (synch and asynch) and *other* (synch and asynch) conditions. Numbness tended to change more with ownership in the *self* conditions. The change data were normal across the subjects (*p *= 0.98, Shapiro–Wilk test) and tended to significance (*T*_11_ = 1.9, *p *= 0.074, one sample *T*-test on *β*). The numbness was thus higher in the *self*, and tended (but did not reach significance) to depend on the level of reported ownership of the rubber hand. Note that the goal of our above analysis is to see a change in the numbness illusion between the self and other conditions with a change in embodiment. Therefore, we omitted subjects who did not feel any embodiment (whose embodiment score did not change between the synch and asynch conditions) or did not feel the numbness illusion (who reported no change in numbness between synch and asynch brushing of both the self and other conditions). Three subjects were omitted in this regard from the analysis.

While these results seemingly support our hypothesis that motor-sensory predictions are modified during the RHI, in addition to the lack of significantly higher *β* in the *self* conditions, we also observed a possible caveat in the paradigm of Experiment 1. In the *self* condition of Experiment 1, when the subject brushed the rubber hand her/himself, then in addition to the vision of the rubber hand being brushed, the subject also experienced the haptic sensation on the right hand (which holds the brush) due to the brush touching the rubber hand. This additional haptic feeling was absent in the *other* condition when the experimenter brushed the rubber hand, and may be considered to be the cause of the increased numbness. To conclusively show that motor-sensory predictions related to the self-generated motor commands are the reason for the increased numbness in the *self* condition, we designed a second experiment in which we equalized the haptic sensation in the right (brushing) hand between the *self* and *other* conditions.

## 3. Experiment 2

**Figure 2.**
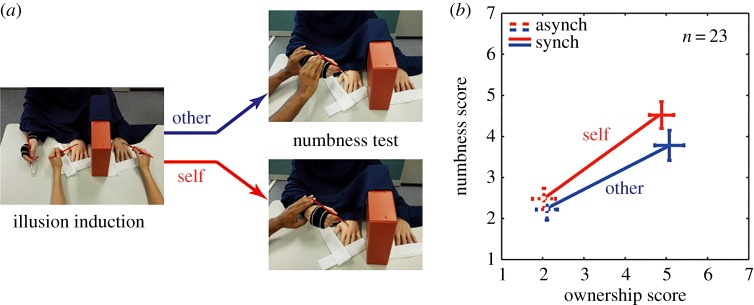
Experiment 2. (*a*) The subjects wore two elastic wrist bands (black bands in figure) on their right hand through this experiment and held a brush in their right hand. As in Experiment 1, the experimenter started each condition by brushing the left hand of the subjects and a rubber hand, either synchronously or asynchronously. This was followed by the numbness test; subjects either relaxed while the experimenter picked their right hand and brushed the rubber hand (other condition), or brushed the rubber hand themselves, while the experimenter kept a light touch on their hand (self condition). (*b*) The numbness score and the ownership score on the seven-point Likert scales plotted together. A 2 × 2 ANOVA revealed that, while the ownership did not change between the *self* and *other* conditions, the numbness changed significantly, *F*_1,88_ = 5.46, *p *= 0.02.

Experiment 2 involved 23 new subjects and used the same set-up, conditions and time-line as Experiment 1. The illusion induction in Experiment 2 was performed with a procedure similar to Experiment 1, by an experimenter brushing the rubber hand and the real left hand of a subject. However, both the *self* and *other* conditions of Experiment 2 required the subjects to hold a brush in their right hand (figure 2*a*). In the *other* condition, the rubber-hand brushing (after the illusion onset) was achieved by a second experimenter lifting the subject's right hand and brushing the rubber hand (see ‘numbness test’ in figure 2*a*) with the brush held in the subject's hand. In the *self* condition, the subjects performed the rubber-hand brushing themselves, while the experimenter maintained a light touch on their right hand at the same location as the *other* condition. The haptic sensation in the right hand due to the brush touching the rubber hand was thus equalized between the *self* and *other* conditions.

Again, we compared the feeling of ownership and numbness across the experiment in a 2 × 2 within subject ANOVA like in Experiment 1. As in the first experiment, some groups of data were not normally distributed (Shapiro–Wilk test of normality *p* ≤ 0.05). Thus, we used the ART followed by the ANOVA. We observed a significant main effect of the visuo-tactile synchrony, *F*_1,88_ = 101.02, *p < *0.0001, but not the brushing type, *F*_1,88_ = 0.554, *p *= 0.458, on the ownership. No interaction was observed between the two factors, *F*_1,88_ = 0.079, *p *= 0.782. Thus, again the RHI increased during synchronous brushing but was similar between the *self* and *other* conditions. Next for numbness, again some data groups were not normally distributed (Shapiro–Wilk test of normality *p* ≤ 0.05) and hence we applied the ART before conducting the ANOVA. The numbness was again observed to suffer from a significant main effect for both visuo-tactile synchrony, *F*_1,88_ = 33.035, *p *<* *0.0001, and brushing type, *F*_1,88_ = 5.46, *p *= 0.02. Once again the interaction between the two factors did not reach significance, *F*_1,88_ = 0.804, *p *= 0.372, but we observed that the numbness change per unit ownership change (*β*) was more in the *self* (1.11 + 0.21 s.e. arb. units) than the *other* (0.67 + 0.18 s.e. arb. units) conditions. The difference was normally distributed across the subjects (*p *= 0.45, Shapiro–Wilk test) and significant (*T*_17_ = 2.337, *p *= 0.031, one sample *T*-test on *β*, see distribution in the electronic supplementary, figure S2). Again five subjects (two who did not show an increase of embodiment after synch compared with the asynch brushing, and three who reported no change in numbness between synch and asynch brushing of both the self and other conditions) were omitted from this analysis. Numbness was thus not only higher when brushing was self-generated (as shown by the ANOVA), but also increased more with ownership across the *self* conditions, compared with the *other* conditions.

## 4. Discussion

Previous studies have repeatedly exhibited an interaction between the sense of ownership and the sensory processes in the brain; multi-sensory integration is considered to be key for self-attribution and the illusion of ownership is believed to modify multi-sensory predictions related to the *owned* limb. It is known that the RHI can be elicited with active movements instead of touches applied by the experimenter [25–27] and that ownership is affected by agency [28]. These observations are attributed not to motor commands generating the motion but to the dynamic proprioceptive and haptic sensations induced by the motion, which substitute the tactile input from the brush strokes. RHI has also been shown to induce changes in our body representations, both the *body image* that is utilized for perception [29,30] as well as the *body schema*, that is used for motor actions [30–32], such as during tool use [33–35].

Our results suggest that ownership additionally modifies the haptic sensory predictions associated with the motor commands generated by the brain. In our preliminary studies, we observed that participants perceive an illusory feeling of numbness when the rubber hand is brushed alone, after the induction of the RHI. Across the subjects (presented in this study and those we used in preliminary studies), numbness was perceived by roughly 75% of the subjects. We show that the numbness illusion is increased when the brushing is performed by oneself, or when there are self-generated movements, compared to when the brushing is done by another.

Motor-sensory predictions by forward models are known to be a fundamental component of the motor system, involved in online motor control [17] and motor learning [18], and that enables human interactions with the self and environment [36], and possibly also with other individuals [37,38]. Our data thus provide evidence to suggest that the effects of illusory ownership on the motor system may be more wide ranging than previously known. With regard to the neural correlates of ownership, previous neuroimaging studies have observed blood oxygenation level-dependent activity in the ventral premotor and cerebellar areas associated with subjective self-attribution of limbs [2,39–41]. These activations have been associated with multi-sensory integration. However, our results suggest the observed premotor and cerebellar activations, which have been previously associated with the loading of an internal model [42] and sensory predictions of self-generated movements [21], may partially be the result of modifications of forward models during the RHI. Furthermore, our result supports a recent suggestion of an action based coding of the body model for self-attribution [43] and is encouraging for embodiment applications in therapy [44] and prosthetics [41] as it shows again that illusions of embodiment and the resulting ownership can be utilized to rapidly influence not just the sensory but also the motor control processes in an individual's brain.

On the other hand, we note here that the change in haptic perception (specifically numbness) observed in our study may be attributed not to a change in the forward model, but a possible increase in *strength* of the multi-sensory predictions associated with the embodied rubber hand (like in [10]) in the presence of self-generated brushing, compared to brushing by others. However, to the best of our knowledge, there is no evidence for the presence of such motor induced, multi-sensory prediction modulation in previous literature. On the other hand, the numbness change agrees with multiple reports of decreased sensory perception during self-generated actions due to the attenuation of the real sensory signals by predictions by the forward model [19,22,23,45]. The fact that in our study these perceptual changes occur when the rubber hand is self-touched suggests that the RHI rewires the forward model such that we expect haptic consequences from the rubber hand. It is though not clear how motor-sensory predictions for the real hand are modified by the RHI; whether the motor-sensory mappings corresponding to the real hand are maintained in parallel to new mappings to the rubber hand, or slowly lost. Recent observations of skin temperature decrease in the real hand during RHI [46] would suggest the latter to be true. But further studies are required to clarify this issue.

## Supplementary Material

Title - Supplementary material for "Forward modeling the rubber hand: illusion of ownership modifies motor-sensory predictions by the brain" Deatails: supplementary methods and results

## Data accessibility

The datasets supporting this article are available at the Dryad Digital Repository: http://dx.doi.org/10.5061/dryad.519qs.
