# Supplementary material for: Forward modelling the rubber hand: illusion of ownership modifies motor-sensory predictions by the brain
Source: R Soc Open Sci. 2016 Aug 24;3(8):160407. doi: 10.1098/rsos.160407 (PMC5108970; doi:10.1098/rsos.160407)
Supplement: Title - Supplementary material for "Forward modeling the rubber hand: illusion of ownership modifies motor-sensory predictions by the brain" Deatails: supplementary methods and results [file rsos160407supp1.docx]

**Supplementary material**

**For**

**Forward modeling the rubber hand: illusion of ownership modifies motor-sensory predictions by the brain**

**Authors:** Laura Aymerich-Franch, Damien Petit, Abderrahmane Kheddar, Gowrishankar Ganesh

*Participants*

In both studies, participants gave their written informed consent prior to participating. They were naïve to the purpose of the experiment. Participants were recruited through a call for volunteers in the website of the experiment and received 1500JPY (Japanese Yen) to participate. Working in the Neuroscience or Psychology fields was used as exclusion criteria. The study was conducted with ethical approval of the National Institute of Advanced Industrial Science and Technology (AIST) in Tsukuba, Japan. A pretest with five volunteers among intern master students and other researchers not connected to the study was carried out for each experiment.

Experiment 1 involved fifteen participants of different nationalities (5 females and 10 males), aged 21 to 37 (M=26.14, SD=4.42). All of them were right handed.

Experiment 2 involved twenty-three participants of different nationalities (15 females and 8 males), aged 20 to 42 (M=27.63, SD=5.91). Two of the participants were left-handed and the rest were right-handed. All participants worked with a left rubber hand.

*Material*

Highly human-looking female and male left rubber gloves [1] were used to develop the rubber hands for the experiment (Figure S1). A surgical elastic glove filled with air and some tissues were introduced in the rubber glove to give volume to the ‘rubber hand’. Hands were assigned according to the gender of the participant. Since the rubber hands did not have nails, the fingertips of the rubber hand as well as the participant’s left hand nails were covered with a tissue in order to maximize the similarity to the real hand. In addition, a small piece of tape was taped between the middle finger and the table so the hand did not move during stroking.

Paint brushes used in Experiment-1 had synthetic bristles. Two size eight paint brushes were used for brushing the participant’s hand to induce the illusion while the subsequent touch of the rubber hand was performed with a size twelve brush. We shifted to horse hair bristles in Experiment-2 after some participants complained to sustained feeling of touch/irritation from the synthetic bristles. Brush sizes were kept same as in Experiment-1.

A 1×1.5m dark thin cotton blanket was used to cover both arms and torso of the participants as well as part of the cardboard that separated the rubber hand from the real left hand and the end of the rubber hand so it was not visible. Both the real right hand and the left rubber hand were left uncovered making sure that the participant was able to see them. The cardboard separator however ensured that the participants did not see their real left hand.

Experiment-2 incorporated two tight fitting elastic wristbands that participants wore on the wrist and four fingers (except thumb) on their right hands as shown in figure 2 in the main text.


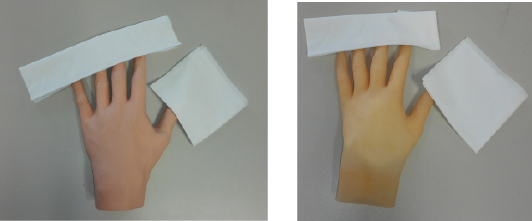


Figure S1. Male and female rubber hands (rubber gloves filled with tissue paper) used in the experiments

*Procedure*

Experiment-1. After reading and signing the consent form, participants were instructed to sit on a chair in front of a table. They placed their right hand on the table in front of them, next to a rubber left hand, and their real left hand behind the orange cardboard separator (Figure 1A in the main text). One of the experimenter sat in front of them on the opposite side of the table and held a paintbrush on each hand. The subjects experienced four conditions: synchronous and asynchronous touch, combined with either *self* or *other* touch. Each subject experienced every condition only once. The pair of *self* (*self*-synch and *self*-asynch) or pair of *other* (*other*-synch and *other*-asynch) conditions were presented consecutively to every subject. However, the order of the *self* and *other* condition pairs was randomized across subjects. Within each pair, the sync and asynch brushing conditions were again randomized across subjects.

The experimenter sitting in front of the participant started each condition brushing the participant’s left hand and the rubber hand either synchronously or asynchronously for 90 seconds, applying constant strokes every 1.5 seconds approximately. The brush strokes were applied (not at one location but) on all the fingers and back of the hand, in a random order such that the same finger or hand area, on both the rubber hand and the real left hand, were stroked at any time in the synch conditions. The brush strokes in the asynch conditions were asynchronous both, in stroke time and in terms of stroked area. After the 90 seconds, a second experimenter asked the three *ownership questions* to participants in order to evaluate the feeling of ownership of the rubber hand. Following the questionnaire, the brushing was continued for 30 more seconds after which the experimenter stopped brushing the real hand and brushed only the rubber hand in the *other* conditions, or stopped brushing both hands and participants picked up a paintbrush (placed near them) with their right hand and brushed the rubber hand themselves. Participants were asked to make brushing strokes similar to the experimenter during the illusion induction such that the rubber hand brushing was similar in frequency and stroke-length across conditions. In order to control the force applied by the subjects during the brushing, we asked the subjects not to ‘push’ but only ‘brush’ the rubber hand and assumed that, as the bristles are compliant, the force cannot exceed the force required to bend the bristles (similar to a hypothesis used also for the popular tactile perception tests with the von-Frey hairs [2]). After the initial brushing for RHI induction, and while they kept seeing the rubber hand being brushed or brushed it, subjects were asked about their perceived *numbness* (explained as the hand feeling sense-less, or ‘dead’) as well as the sensory illusions of *tickling* and *touch.* We used these questions that represent opposite sensations because initially, while we expected a change in haptic perception during self-generated brushing, we were unsure if the changed perception would be described as numbness or also as tickling or touch. In pretests we observed that subjects mostly reported a feeling of numbness, but we retained the questions on tickling and touch as one subject reported feeling both numbness and touch in the pretest. After finishing each condition, participants were given a 30-second break. Before starting the following condition, in order to nullify the illusion from the previous condition, subjects were asked to move and look at their real left hand and one of the experimenters touched their left hand (a female experimenter touched their hand if the participant was female and a male experimenter touched their hand if the participant was male). After completing the experiment, participants were asked to verbally share their thoughts about the experience and the experimenters explained them more details about the rubber hand illusion paradigm. Before leaving, participants were asked about the necessary details to proceed with the payment and thanked for their participation.

Experiment-2. The setup, procedure, and experimental conditions were similar to Experiment-1. As a substantial difference, participants held a paint brush in their right hand in all conditions (*self* and *other*) as shown in figure 2A in the main text. In addition, participants were asked to wear two wristbands, one on their wrist and another over four of their fingers (except the thumb) on their right hand. After the illusion-induction phase of 90 seconds, participants again answered the ownership questionnaire, and then as in Experiment-1, the brushing continued for 30 more seconds. Following this, in the *other* condition, a second experimenter lifted the subjects’ right hand by holding their hand over the wrist bands, and brushed the rubber hand as shown in figure 2B in the main text. During this process, participants were asked to leave their arm weight on the experimenters hand and to not make any movement with the hand. In the *self* condition, subjects used the paint brush they held to actively perform the rubber hand brushing. At the same time, the experimenter maintained a light touch on the wristband worn by the participant on the right hand, at the same location where she held the participants’ hand in the *other* condition (figure 2C in the main text). The elastic wrist bands thus served two purposes. One, they attenuated the difference in the touch and clasp the subjects felt in the *other* and *self* conditions and, second, they ensured the experimenter supported the subject hand at the same location in each condition. The haptic sensation in the right hand due to the brush touching the rubber hand was thus equalized between the *self* and *other* conditions. After the brushing, participants verbally reported their feeling of *numbness*, *tickling*, and *touch*. Following which, the experiment continued until completing the four conditions similar to Experiment-1.

*Measures*

The embodiment questionnaire was adapted from [2] and consisted of three questions (*Do you feel as if…*

*1. the touch of the paintbrush is in the location of the rubber hand;*

*2. the touch you feel is caused by the paintbrush touching the rubber hand;*

*3. the rubber hand is your hand*).

One of the experimenter asked the questionnaire out loud to the participants. The participants verbally reported the answers for each of the three statements which were rated on a 7-point Likert scale that ranged from *not at all* to *very strongly*.

After 30 seconds of further brushing, participants were asked out loud whether they were feeling numbness in their real hand. Participants verbally reported the feeling rating it on a 7-point Likert scale that ranged from *not at all* to *very strongly* while they were experiencing the brushing in the rubber hand only. In addition, participants were also asked whether they felt tickling or the touch of the paintbrush in their real hand, again on a 7-point Likert scale that ranged from *not at all* to *very strongly*,

Before starting the experiment participants were informed that all questions in the questionnaire were regarding their left hand.

At the end of the experiment participants were explained about the general purpose of the experiment and the well-known rubber hand illusion. They were also asked to provide information about gender and age.

*Supplementary Results*

The means of the three questions regarding embodiment were averaged in each condition to obtain the embodiment score. Table 1 shows mean and SD for each experimental condition in experiment 1 and 2.

**TableS1. Mean (SD) for embodiment by experimental condition for experiment 1 and 2**

|  | Synchronous | | Asynchronous | |
| --- | --- | --- | --- | --- |
|  | Self | Other | Self | Other |
|  |  | Experiment-1 |  |  |
| Q1 | 6(1.13) | 5.93(1.66) | 2.27(1.38) | 1.93(1.62) |
| Q2 | 6(1.06) | 5.47(1.88) | 1.93(1.44) | 1.93(1.48) |
| Q3 | 5.13(1.24) | 4.53(1.84) | 1.93(1.33) | 1.93(1.57) |
| Global | 5.71(.93) | 5.31(1.63) | 2.04(1.06) | 1.93(1.19) |
|  |  | Experiment-2 |  |  |
| Q1 | 5.04(1.71) | 5.34(1.92) | 1.95(1.55) | 1.87(1.21) |
| Q2 | 5.13(1.68) | 5.08(1.75) | 1.95(1.36) | 2.21(1.78) |
| Q3 | 4.47(1.47) | 4.78(1.83) | 2.17(1.49) | 2.21(1.31) |
| Global | 4.88(1.44) | 5.07(1.71) | 2.03(1.36) | 2.10(1.24) |

For the statistical analysis, we utilized a 2×2 ANOVA. The first factor was the visuo-tactile brushing synchrony which had two levels, synchronous and asynchronous. The second factor was brushing type, which was either *other* or *self.* A separate ANOVA was used for embodiment and numbness respectively. Since some groups of data did not reach a normal distribution (Shapiro-Wilk test of normality p<.05) we used the Aligned Rank Transform (ART) for nonparametric factorial data alignment [4] before a 2X2 ANOVA. The ART relies on a preprocessing step that “aligns” data before applying averaged ranks, after which common ANOVA procedures can be performed.

Additionally, we checked for differences in tickling and touch. As expected, for Experiment-1, the two-way within-subjects ANOVA did not reveal a significant main effect for brushing type on tickling *F*(1,56)=.019, *p*=.889, or touch *F*(1,56)=.233, *p*=.631. Similarly, for Experiment-2, the two-way within-subjects ANOVA did not show a significant main effect for brushing type on tickling *F*(1,88)=.038, *p*=.846, or touch *F*(1,88)=.086, *p*=.77.

**TableS2. Mean (SD) for numbness, tickling, and touch by experimental condition for experiment 1 and 2**

|  | Synchronous | | Asynchronous | |
| --- | --- | --- | --- | --- |
|  | Self | Other | Self | Other |
|  |  | Experiment-1 |  |  |
| Numbness | 4.53(2.50) | 3.00(2.33) | 2.66(2.26) | 2.20(1.97) |
| Tickling | 2.20(1.47) | 2.46(2.19) | 1.93(1.38) | 1.93(1.43) |
| Touch | 2.33(2.16) | 2.46(1.99) | 1.46(1.55) | 1.60(1.05) |
|  |  | Experiment-2 |  |  |
| Numbness | 4.52(1.59) | 3.78(1.78) | 2.47(1.27) | 2.21(1.27) |
| Tickling | 1.69(1.18) | 1.82(1.37) | 1.47(.89) | 1.30(.70) |
| Touch | 2.08(1.41) | 2.13(1.45) | 1.39(.65) | 1.30(.55) |

*
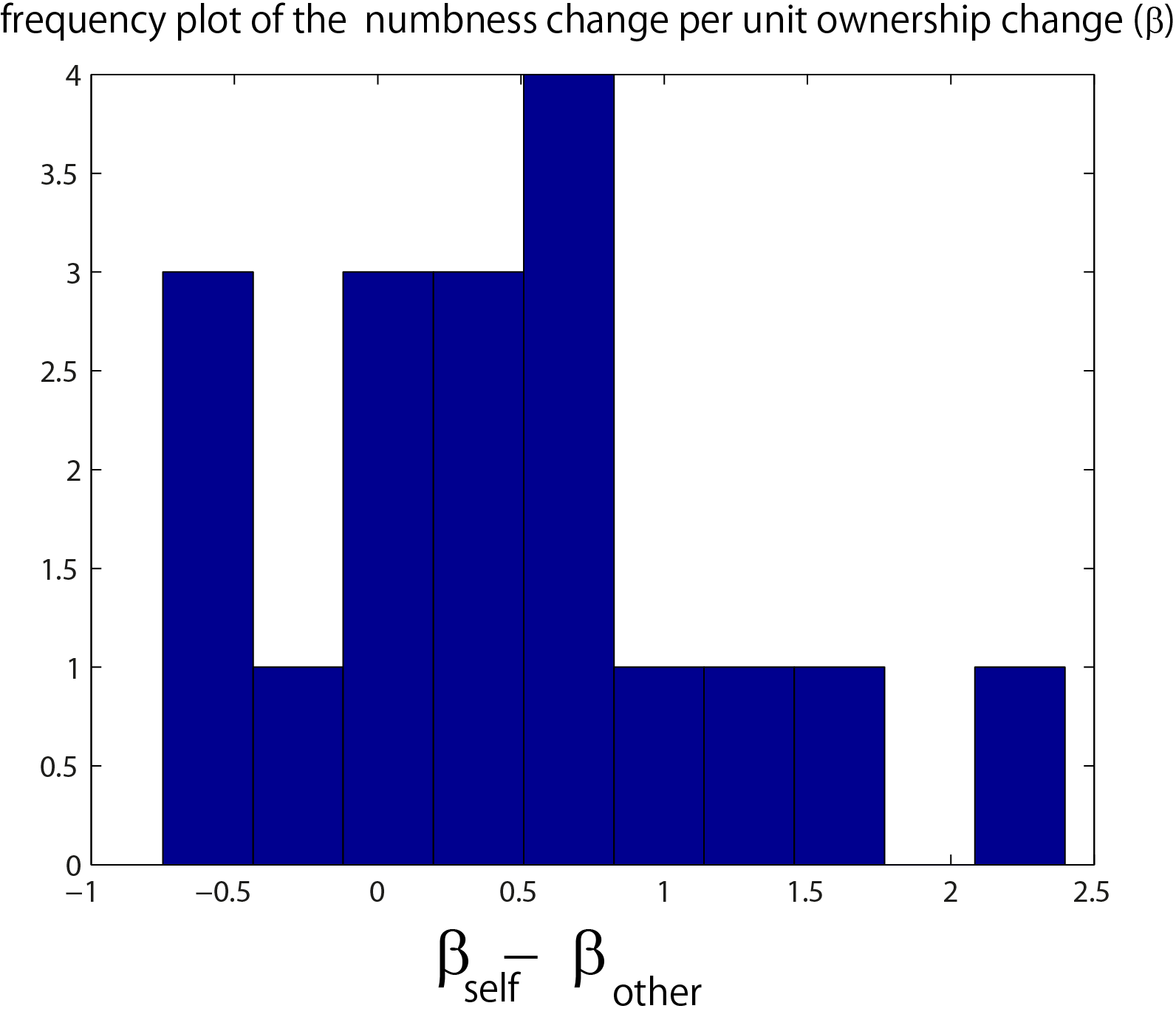
*

**Figure S2: Data distribution of the difference of change per unit ownership change (β) between the self and other conditions in Experiment-2 was significantly more than zero (T(17)=2.337, *p*=.031, one sample T-test on β.**

*Numbness change per unit ownership change*

In order to ascertain how change in embodiment influences numbness perception in individual subjects, we modelled the numbness change (ΔN) between the asynch and synch conditions to depend linearly on the ownership change (ΔO) between the same two conditions; ΔN=β×ΔO. We then calculated the *numbness change per unit ownership change* (given by β) for each subject and compared how β differed between the *self* (synch and asynch) and *other* (synch and asynch) conditions. The difference in β tended to significance (T(11)=1.97, p=.074, one sample T-test on β) in Experiment 1 but was significant in Experiment 2 (T(17)=2.337, *p*=.031, one sample T-test on β). Fig. S2 shows the distribution of subject differences from Experiment 2.

*References*

1. Jiang, Y. et al. 2014. Development and evaluation of simplified EMG prosthetic hands. Proceedings of the 2014 IEEE International Conference on Robotics and Biomimetics, pp.1368-1383, Bali, Indonesia, Dec. 5-10.
2. Lambert, G. A., Mallos, G. & Zagami, A. S. 2009. Von Frey’s hairs--a review of their technology and use--a novel automated von Frey device for improved testing for hyperalgesia. J. Neurosci. Methods. 177, 420–426. (doi:10.1016/j.jneumeth.2008.10.033)
3. Botvinick M, Cohen J. 1998. Rubber hands ‘feel’ touch that eyes see. Nature391, 756. (doi:10.1038/35784)
4. Wobbrock JO, Findlater L, Gergle D, Higgins JJ. 2011. The Aligned Rank Transform for nonparametric factorial analyses using only ANOVA procedures. In Proc. of the ACM Conference on Human Factors in Computing Systems (CHI ‘11), Vancouver, British Columbia, May 7–12, 2011, pp. 143–146. New York, NY: ACM Press
